# Electric-field control of anomalous and topological Hall effects in oxide bilayer thin films

**DOI:** 10.1038/s41467-017-02629-3

**Published:** 2018-01-15

**Authors:** Yuki Ohuchi, Jobu Matsuno, Naoki Ogawa, Yusuke Kozuka, Masaki Uchida, Yoshinori Tokura, Masashi Kawasaki

**Affiliations:** 1 Department of Applied Physics and Quantum-Phase Electronics Center (QPEC), University of Tokyo, Tokyo, 113-8656 Japan; 2 RIKEN Center for Emergent Matter Science (CEMS), Wako, Saitama 351-0198 Japan

## Abstract

One of the key goals in spintronics is to tame the spin-orbit coupling (SOC) that links spin and motion of electrons, giving rise to intriguing magneto-transport properties in itinerant magnets. Prominent examples of such SOC-based phenomena are the anomalous and topological Hall effects. However, controlling them with electric fields has remained unachieved since an electric field tends to be screened in itinerant magnets. Here we demonstrate that both anomalous and topological Hall effects can be modulated by electric fields in oxide heterostructures consisting of ferromagnetic SrRuO_3_ and nonmagnetic SrIrO_3_. We observe a clear electric field effect only when SrIrO_3_ is inserted between SrRuO_3_ and a gate dielectric. Our results establish that strong SOC of nonmagnetic materials such as SrIrO_3_ is essential in electrical tuning of these Hall effects and possibly other SOC-related phenomena.

## Abstract (web summary)

Spintronic devices are typically controlled with magnetic fields or current injection but incorporating these mechanisms into high-density devices is difficult. Here the authors present an oxide device where the topological and anomalous Hall effects can instead be modulated using an electric field.

## Introduction

Profound implications for the electric field control of spin states have been revealed through magneto-transport properties arising from the spin-orbit coupling (SOC)^1^. Such properties have been attracting great interest as foundations for high-density and low-power-consumption spintronic devices because the properties coupled to the spin states enable spin manipulation without magnetic field variation or large current injection. Among the magneto-transport processes, of interest here is the intrinsic anomalous Hall effect^2^ (AHE), which is related to magnetization (*M*) in Hall resistivity (*ρ*_AHE_ = *R*_S_*M*). In some itinerant ferromagnets such as SrRuO_3_^3, 4^, the proportionality factor (*R*_S_) is governed by *k*-space monopoles, i.e., singularities originating from band-crossings gapped by SOC; not *M* but *R*_S_ is potentially tuneable by electric field there. Another intriguing example is the topological Hall effect (THE) originating from scalar spin chirality concomitant with non-coplanar spin structures such as in a frustrated pyrochlore magnet^5^ and in metallic magnets characterized by a non-zero skyrmion number (*N*_sk_)^6–9^. In the case of *N*_sk_ = 1, the topologically protected spin swirling texture is called magnetic skyrmion^6, 10, 11^. Despite mounting interest in electrical control of these Hall effects, however, their control in itinerant magnets has been elusive in conventional field-effect structures, that is, magnetic materials adjacent to gate dielectrics. Recent discovery of the THE in an oxide heterostructure composed of SrRuO_3_ and SrIrO_3_^12^ brings an opportunity to develop electrical control because the strong SOC in SrIrO_3_^13^ induces a SOC-related phenomenon in neighboring ferromagnetic SrRuO_3_. In addition to the intrinsic AHE inherent to SrRuO_3_^3^, the combination of the strong SOC and the broken inversion symmetry gives rise to substantial Dzyaloshinskii–Moriya interaction (DMI)^6, 14^ and the concomitant THE; the underlying spin texture has been deduced to be the interface-driven skyrmion^12^. In view of the fact that ultrathin films of itinerant magnets have been a playground for electrical manipulation of spin states^1, 15–19^, an electric field applied to the ultrathin heterostructure may provoke effective manipulation of the spin state contending with screening effects by itinerant electrons of SrRuO_3_. Furthermore, we can expect that the strong SOC in SrIrO_3_ plays a positive role at the interface as well because both AHE and THE are magneto-transport phenomena driven by SOC.

Here, we show the clear modulation of both AHE and THE by an electric field in the heterostructure of SrRuO_3_/SrIrO_3_/SrTiO_3_ from magneto-transport and magneto-optic Kerr effect (MOKE) measurements. The electrical modulation is effective only when SrIrO_3_ is inserted between SrRuO_3_ and a gate dielectric, i.e., SrTiO_3_. This indicates the essential role of the strong SOC in nonmagnetic materials for the electrical tuning of these Hall effects and possibly other SOC-related phenomena.

## Results

### Stacking-order dependent electric field control

**Fig. 1:**
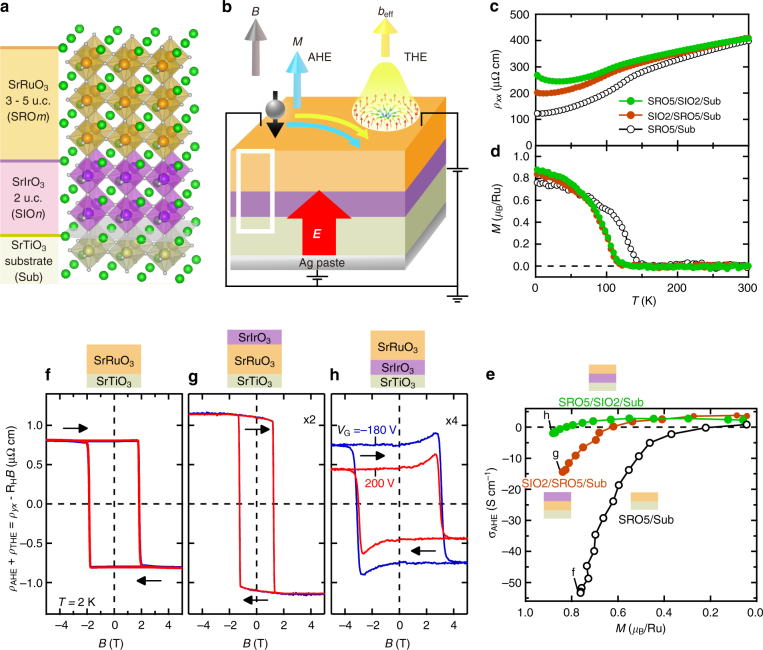
Structure and basic physical properties of samples. **a** Schematics of the SrRuO_3_-SrIrO_3_ bilayer film, where SrIrO_3_ is inserted between SrRuO_3_ and SrTiO_3_ substrate (SRO*m*/SIO2/Sub, *m* = 3–5 unit cells). Green, orange, purple, beige, and gray atoms represent Sr, Ru, Ir, Ti, and O, respectively, in the crystal structure, which is drawn using VESTA^38^. **b** Schematics of the magneto-transport properties observed in SRO*m*/SIO2/Sub under the application of a gate electric field (*E*): Anomalous Hall effect (AHE) generated by magnetization (*M*) and topological Hall effect (THE) driven by fictitious magnetic field (*b*_eff_) in the case of skyrmion formation. White box corresponds to the schematics in **a**. Temperature (*T*) dependence of longitudinal resistivity (*ρ*_*xx*_, **c**) and out-of-plane magnetization measured at 0.1 T (*M*, **d**) for SRO5/Sub, SIO2/SRO5/Sub, and SRO5/SIO2/Sub. **e** Anomalous Hall conductivity (*σ*_AHE_) as a function of magnetization (*M*). **f**–**h** The sum of anomalous and topological Hall resistivity (*ρ*_AHE_ + *ρ*_THE_) at 2 K as a function of external magnetic field (*B*) under application of gate bias *V*_G_ = − 180 V (blue lines) and 200 V (red lines). At the lowest temperature (*T* = 2 K), the largest modulation of carrier density can be realized due to the quantum paraelectric nature of SrTiO_3_ substrates. On top, respective sample structure is shown. *ρ*_AHE_ + *ρ*_THE_ is deduced by subtracting a *B*-linear ordinary Hall component (*R*_H_*B*) from the Hall resistivity (*ρ*_*yx*_). Black arrows indicate the sweep direction of *B*

Five-unit-cell SrRuO_3_ thin films were epitaxially grown on SrTiO_3_(001) substrates by pulsed laser deposition in three stacking orders with SrIrO_3_ (see Methods): a single layer of SrRuO_3_ (SRO5/Sub), a bilayer of SrRuO_3_ and 2-unit-cell SrIrO_3_ (SIO2/SRO5/Sub), and that with the inverted deposition order (SRO5/SIO2/Sub, Fig. 1a). We first take a look at basic transport and magnetic properties before we go into the Hall effects under an applied electric field (Fig. 1b). In Fig. 1c, d, we show the temperature dependence of longitudinal resistivity (*ρ*_*xx*_) and magnetization perpendicular to the film plane in the three samples. All of them show metallic conduction and ferromagnetic magnetization whose Curie temperatures (*T*_C_) are lower than the bulk value (160 K)^3^. These transport and magnetic properties are consistent with previously reported ultrathin SrRuO_3_ films^20^. Figure 1e shows anomalous Hall conductivity (*σ*_ΑΗΕ_) as a function of magnetization with temperature as a control parameter. The *σ*_AHE_ of the three samples behave the same way; when the magnetization gets larger, the sign of *σ*_AHE_ is inverted from positive to negative. This sign inversion is consistent with the previous experiments^4, 12^, manifesting the above-mentioned band anti-crossing singularities of SrRuO_3_. Conversely, such temperature dependence in AHE implies the potential for electrical control of AHE through the modification of the SOC-induced band.

In contrast to the similarities of the basic transport and magnetic properties among the three samples, a clear difference emerges in electric field effect (see Methods). The sums of anomalous and topological Hall resistivities (*ρ*_AHE_ + *ρ*_THE_) under applied electric fields are shown in Figs. 1f–h as a function of external magnetic field (*B*). The *B* dependence of *ρ*_AHE_ corresponds to that of magnetization normal to the film plane, whereas *ρ*_THE_ shows up with the formation of the topological spin texture as exemplified by the peak at around 3 T in Fig. 1h. The sum of these Hall components is obtained by subtracting the *B*-linear ordinary Hall term (*R*_H_*B*) from the Hall resistivity (*ρ*_*yx*_). Both in SRO5/Sub (Fig. 1f) and in SIO2/SRO5/Sub (Fig. 1g), *ρ*_AHE_ +* ρ*_THE_ is almost unchanged by the applied electric field. However, *ρ*_AHE_ +* ρ*_THE_ is obviously changed in SRO5/SIO2/Sub (Fig. 1h). This striking stacking-order dependence suggests that the electric field to the SrRuO_3_-SrIrO_3_ interface causes the noticeable modulation. The thinness of the SrIrO_3_ layer compared with the SrRuO_3_ layer contributes to the preferable modulation in SRO5/SIO2/Sub because the interface in SRO5/SIO2/Sub is closer to the gate dielectric than that in SIO2/SRO5/Sub (see Supplementary Figure 1 and Supplementary Note 1). Hereafter, we focus on the control in the SRO5/SIO2/Sub sample.

### Electric-field modulation of AHE

**Fig. 2:**
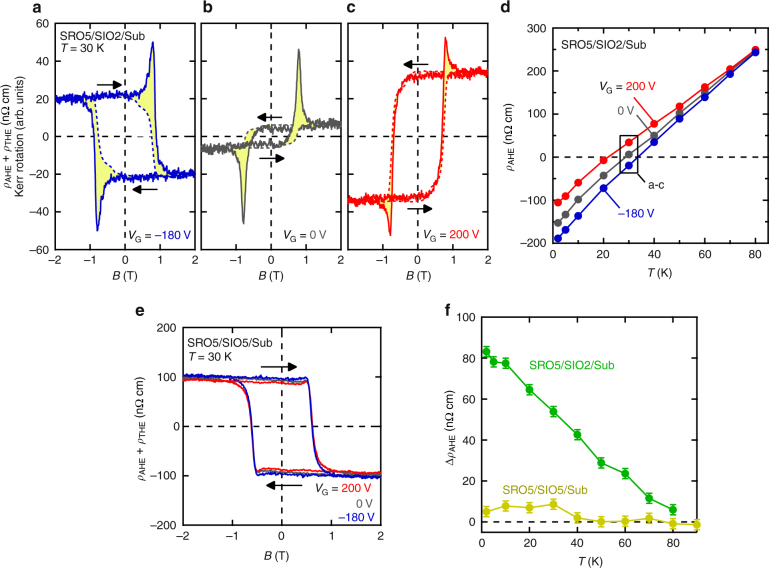
Electric-field control of anomalous Hall effect. Magnetic-field (*B*) dependence of anomalous and topological Hall resistivities (*ρ*_AHE_ + *ρ*_THE_, solid lines) at 30 K under gate voltage *V*_G_ = −180 V (**a**), 0 V (**b**), and 200 V (**c**) for SRO5/SIO2/Sub. Magneto-optic Kerr rotation as a function of *B* under the same gate bias is also shown by broken lines in each panel. Yellow colored regions correspond to *ρ*_THE_. Black arrows indicate the sweep direction of *B*. **d** Temperature (*T*) dependence of *ρ*_AHE_ under *V*_G_ = −180 V (blue), 0 V (gray) and 200 V (red). The black box corresponds to the data in **a**–**c**. **e** *B* dependence of *ρ*_AHE_ + *ρ*_THE_ at 30 K under *V*_G_ = −180 V (blue), 0 V (gray), and 200 V (red) for SRO5/SIO5/Sub. **f** *T* dependence of variation in anomalous Hall resistivity (Δ*ρ*_AHE_) between *V*_G_ = 200 V and *V*_G_ = −180 V for SRO5/SIO5/Sub (yellow) and SRO5/SIO2/Sub (green). Error bars include the uncertainty of the sample thickness and of electrical measurement

In the structure of SRO5/SIO2/Sub, the large electric field modulation gives rise to the sign inversion of AHE without changing temperature. Magnetic-field dependences of *ρ*_AHE_ + *ρ*_THE_ under different electric fields at 30 K are shown in Fig. 2a–c. Except for the topological Hall term that shows a peak at around 0.8 T, the data are dominated by the anomalous Hall contribution. In particular, we can consider the value at high magnetic field such as 2 T to be totally derived from *ρ*_AHE_ since all the spins are ferromagnetically aligned without forming any topological spin textures. The sign of *ρ*_AHE_ above the saturation field is inverted from negative to positive when the gate voltage is varied from negative (−180 V) to positive (200 V), while *ρ*_AHE_ is close to vanish under zero bias. This sign inversion indicates the sign reversal of the proportionality factor *R*_S_ because MOKE measurements reveal that only a minor fraction of *M*, <10% of the magnetization, is electrically modulated (see Supplementary Figure 2 and Supplementary Note 2). Such electrical sign inversion has never been observed in plain films of itinerant magnets including SrRuO_3_.

As shown in Fig. 2d, the tendency of AHE change is nearly temperature independent; the positive (negative) bias voltage increases (decreases) *ρ*_AHE_ regardless of the sign of AHE. Since singularities in the band structure of SrRuO_3_ originate from band anti-crossings gapped by SOC, we speculate that the electric field control of AHE is ascribed to the redistribution of the singularities caused by the variation of SOC. The pronounced controllability in SRO5/SIO2/Sub compared with its absence in SIO2/SRO5/Sub suggests that the electric field from SrIrO_3_ side induces certain modification of SOC in the bilayer even if it has itinerant carrier density as high as 10^22^ cm^−3^ in the magnetic SrRuO_3_ layer. This is in sharp contrast to the case of chemically doped EuTiO_3_^21^, where carrier-density variation inverts the sign of AHE accompanied by the shift of Fermi energy (*E*_F_). The insertion of the SrIrO_3_ layer makes a more important contribution to the AHE modulation than the *E*_F_ shift in SrRuO_3_ does. The controllability may partly rely on the lower carrier density of inserted semimetallic SrIrO_3_, the order of 10^19^ cm^−3^ ^22^.

In order to clarify the effectiveness of the heterointerface close to the gate dielectric, we also fabricate the heterostructure with thicker (5 unit cells) SrIrO_3_ (SRO5/SIO5/Sub, see Supplementary Figures 3–5 and Supplementary Notes 3 and 4). Figure 2e shows magnetic field dependences of *ρ*_AHE_ + *ρ*_THE_ under different electric fields at 30 K in SRO5/SIO5/Sub, where the change of *ρ*_AHE_ is apparently suppressed. In Fig. 2f, we compare the variation of *ρ*_AHE_ (Δ*ρ*_AHE_ = *ρ*_AHE_ (*V*_G_ = 200 V)−*ρ*_AHE_ (*V*_G_ = −180 V)) in SRO5/SIO5/Sub with that in SRO5/SIO2/Sub, where we can clearly see the suppression of the modulation in the thicker SrIrO_3_ sample within all the measured temperatures. We attribute this suppression to the significant screening of the electric field within the thicker SrIrO_3_ layer.

### Electric-field modulation of THE

**Fig. 3:**
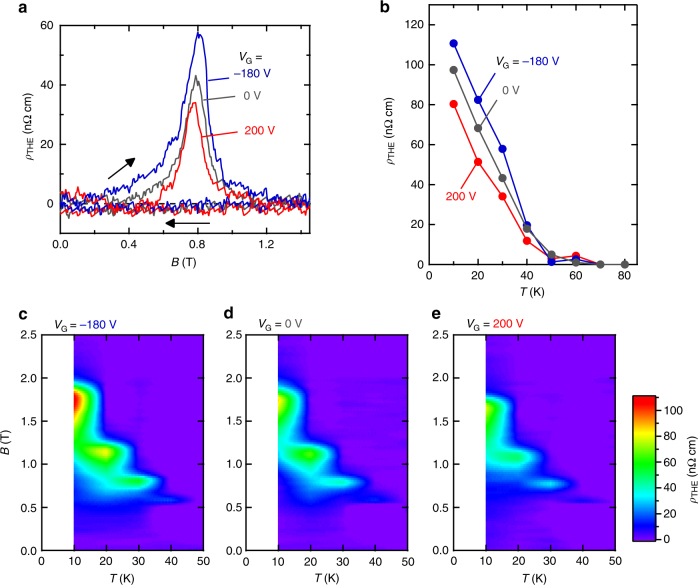
Electric-field control of topological Hall effect. **a** Topological Hall resistivity (*ρ*_THE_) at 30 K as a function of external magnetic field (*B*) under *V*_G_ = −180 V (blue line), 0 V (gray line) and 200 V (red line) for SRO5/SIO2/Sub. Black arrows indicate the sweep direction of *B*. **b** Temperature (*T*) dependence of *ρ*_THE_ under gate bias. Color map of *ρ*_THE_ in the *T*-*B* plane under *V*_G_ = −180 V (**c**), 0 V (**d**) and 200 V (**e**)

THE is also modulated by electric field in the structure of SRO5/SIO2/Sub. In order to evaluate *ρ*_THE_ from magneto-transport measurement, *ρ*_AHE_ has to be subtracted from *ρ*_AHE_ + *ρ*_THE_. Since MOKE is proportional to magnetization as anticipated from its perturbative nature and also as experimentally verified in the previous report^12^, MOKE under applied electric field can be utilized as the reference of *ρ*_AHE_, which is also proportional to magnetization at a constant temperature. The magnetic field dependences of Kerr rotation are shown by the broken lines in Fig. 2a–c. Each Kerr rotation is normalized by *ρ*_AHE_ at high magnetic field under each applied electric field; the normalized curves represent the *B* dependence of *ρ*_AHE_. By subtracting them from *ρ*_AHE_ + *ρ*_THE_, we deduced *ρ*_THE_ (yellow colored regions in Fig. 2a–c). Figure 3a shows the electric field-controlled peak of *ρ*_THE_ around *B* = 0.8 T. The electric field also tunes the range of magnetic field where finite *ρ*_THE_ appears; when the positive (negative) gate voltage is applied, *ρ*_THE_ gets smaller (larger) and the magnetic field region gets shrunk (enlarged). The variation in peak of *ρ*_THE_ (Δ*ρ*_THE_ = *ρ*_THE_ (*V*_G_ = 200 V)−*ρ*_THE_ (*V*_G_ = −180 V)) normalized by zero-bias value (Δ*ρ*_THE_/*ρ*_THE_ (0 V)) is as large as 55%. The sign of THE remains the same during the sign inversion of AHE by electric field, supporting that the origins of AHE and THE are clearly distinct from each other as previously indicated^12^.

Assuming the skyrmion formation in this heterostructure, we discuss the relation of this THE modulation with skyrmion length scale. *ρ*_THE_ is an outgrowth of ordinary Hall effect under fictitious magnetic field (*b*_eff_) caused by the non-coplanar spin texture of skyrmion^6^. Since one skyrmion generates one flux quantum (*Φ*_0_ = *h*/*e*), *ρ*_THE_ is described as a function of skyrmion density (*n*_sk_):

1 $$\rho _{{\mathrm{THE}}} = PR_{\mathrm{H}}b_{{\mathrm{eff}}} = PR_{\mathrm{H}}n_{{\mathrm{sk}}}\Phi _0$$

where *P* is spin polarization of conduction electrons in SrRuO_3_. Ordinary Hall coefficient *R*_H_ is inversely proportional to carrier density. The change of *P* or *R*_H_ is negligible because *E*_F_ in SrRuO_3_ remains almost unchanged judging from the variation of *ρ*_*xx*_ as small as the order of 1% as discussed later. Therefore, the modulation of *ρ*_THE_ indicates the change of *n*_sk_. The skyrmion size, estimated by $r_{{\mathrm{sk}}} \cong 1/\sqrt {n_{{\mathrm{sk}}}}$, is varied from 15 nm (*V*_G_ = 200 V) to 12 nm (*V*_G_ = −180 V) adopting *P* = −9.5% as a typical value of SrRuO_3_^23^ and *R*_H_ evaluated from the *B*-linear component of Hall resistivity under *V*_G_ = 0 V at 30 K. Since *r*_sk_ is proportional to the ratio of ferromagnetic interaction *J* to DMI *D*, or *J*/*D*^6^, the positive (negative) gate voltage should increase (decrease) *J*/*D*. The controlling knob of *ρ*_THE_ is thought to be *D* rather than *J*; the variation of *J* is negligible because the applied electric field hardly varies *T*_C_, which can be evaluated by a kink structure in *ρ*_*xx*_^3^ (see Supplementary Figure 6 and Supplementary Note 5). The real-space observation of such electrical control of skyrmion size would be one of the future challenges.

We examine the other possibilities of the underlying spin texture. One of the candidates is the conical spin structure, which is topologically trivial and is reported to give rise to THE-like signal^24^. In our case, this can be excluded since the spin structure is dominated by conventional ferromagnetic phase with finite anisotropy as exemplified by open hysteresis loops in magnetization (see Supplementary Figure 2). The possibility of non-coplanar spin structures stabilized only under geometrical frustration^5, 25^ can also be excluded because such frustration is apparently absent in our heterostructure. We then focus on a topological spin texture other than skyrmion e.g., biskyrmion or meron, of which topological number (*N*_sk_) is not unity^9, 26, 27^. The emergent fictitious magnetic field can thus be generalized as *n*_topo_*N*_sk_*Φ*_0_, where *n*_topo_ is the density of the topological spin texture. Since *N*_sk_ is constant inherent in the topological spin structures and hence independent of the gate bias, the electric field modulation of *ρ*_THE_ is attributed to the modulation of *n*_topo_. In order to realize topologically nontrivial spin structures in SrRuO_3_, the interface-driven DMI is required as discussed in the above-mentioned skyrmion case. The ratio of *J*/*D* is inversely correlated with *n*_topo_ for any type of topological spin textures. The consequence therefore remains the same; positive (negative) gate voltage should increase (decrease) *J*/*D*.

Considering that the variation of DMI is attributed to the modification of SOC at the interface, both modulations of AHE and THE are brought about by the same origin; this appears in the temperature dependence. The tendency of *ρ*_THE_ change is independent of temperature as shown in Fig. 3b. The expansion of the observable magnetic field region under negative electric field is discerned at every temperature below 40 K (Fig. 3c–e). These indicate that the obtained modulation of THE is independent of the sign of AHE, which is inverted at 30 K. The temperature independent trend of the electric field effect on THE is in accord with that on AHE (Fig. 2d), implying the common origin of SOC variation. In fact, the MOKE measurements indicate that the coercive force (*H*_C_) is shifted from 0.73 T (*V*_G_ = 200 V) to 0.76 T (*V*_G_ = −180 V) at 30 K (see Supplementary Figure 2 and Supplementary Note 2). This shift of *H*_C_ also supports the modification of SOC because the magnetic anisotropy originates from SOC.

## Discussion

**Fig. 4:**
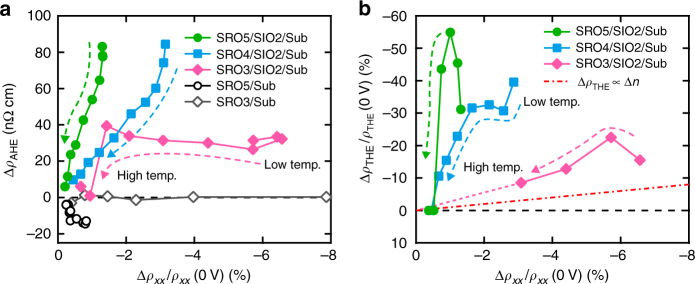
Qualitative analyses of modulations in AHE and THE. **a** Difference in anomalous Hall resistivity (Δ*ρ*_AHE_) between *V*_G_ = 200 V and *V*_G_ = −180 V as a function of longitudinal resistivity variation ratio (Δ*ρ*_*xx*_/*ρ*_*xx*_ (0 V)) at temperatures ranging from 2 K to 80 K. **b** Modulation ratio of topological Hall resistivity (Δ*ρ*_THE_/*ρ*_THE_ (0 V)) as a function of Δ*ρ*_*xx*_/*ρ*_*xx*_ (0 V). Red dash-dotted line is the calculated variation where gate bias only changes carrier density (Δ*n*). Pink dotted line is the guide to the eyes. Broken arrows indicate temperature variation

The observed modulations of the AHE and THE are not simply explained by the variation of carrier density. In order to show this clearly, the variations of AHE (Δ*ρ*_AHE_) and THE (Δ*ρ*_THE_/*ρ*_THE_ (0 V)) with different thickness of SrRuO_3_ films are shown in Fig. 4a, b, respectively, as a function of *ρ*_*xx*_ variation normalized by zero-bias value (Δ*ρ*_*xx*_/*ρ*_*xx*_ (0 V)) at various temperatures (see Supplementary Figures 7–9 and Supplementary Notes 6–8 for detailed transport and magnetic properties). Δ*ρ*_*xx*_/*ρ*_*xx*_ (0 V) ranges from 1.2% in SRO5/SIO2/Sub to 6.6% in SRO3/SIO2/Sub at 10 K. Since *ρ*_*xx*_ is inversely proportional to carrier density (*n*), the order of magnitude is consistent with the expected electron accumulation by a SrTiO_3_-back-gate transistor (Supplementary Note 8). Δ*ρ*_AHE_ in bilayers indicates that the gate-bias control of AHE has the specific tendency regardless of the film thickness. The same plots of SrRuO_3_ single layers (SRO*m*/Sub) are also depicted for comparison. They have negligible change in AHE, while their *ρ*_*xx*_ variation is in the same range with that in bilayers with the same SrRuO_3_ thickness. As already pointed out, these results demonstrate the importance of inserting the SrIrO_3_ layer rather than the *E*_F_ shift in SrRuO_3_. According to the equation (1), *ρ*_THE_ is proportional not only to *n*_sk_ (or *n*_topo_*N*_sk_) but also to *R*_H_. We show the calculated variation of *ρ*_THE_ contributed from *R*_H_, i.e., *n* variation (Δ*R*_H_ i.e. Δ*n* = *n*(*V*_G_ = 200 V) − *n*(*V*_G_ = −180 V)) with the red dash-dotted line in Fig. 4b, assuming small Δ*n* (Δ*R*_H_ ∝ Δ*n*/*n*^2^, i.e., Δ*ρ*_THE_ ∝ Δ*n*) and *V*_G_-independent electron mobility. All the modulations in heterostructures are located far above the red dash-dotted line. Therefore, most of the observed Δ*ρ*_THE_ is attributed to the change in size of the topological spin texture, i.e., DMI. These pronounced modulations of the AHE and THE are both achieved not by carrier-density variation in SrRuO_3_ but by modification of SOC at the interface of SrRuO_3_ and SrIrO_3_.

Recent theoretical calculations^28, 29^ have revealed that the DMI is enormously affected by the singularities in band structures as well as the AHE, although the precise dependencies differ between them. This has been confirmed in itinerant chiral magnets^30^ and a semiconducting Dirac electron system^31^. We speculate that our bilayers have some band deformation when applying an electric field to the interface. In both plots in Fig. 4, thinner SrRuO_3_ films exhibit smaller variation in spite of the larger Δ*ρ*_*xx*_/*ρ*_*xx*_ (0 V). This thickness dependence is probably attributed to band structure deformation as observed in the thin limit of SrRuO_3_^32^; the density of states near *E*_F_ shrinks as SrRuO_3_ gets thinner.

One of the plausible pathways in the electric field-induced change of SOC is through the interface potential gradient, which is observed as the electrical control of Rashba-type band splitting in a semiconductor quantum well^33^. In contrast to the conventional Rashba systems, however, we have to take into account of the strong SOC of SrIrO_3_, the magnetic properties of SrRuO_3_, and their strong hybridization. Furthermore, since the itinerant-electron systems including SrRuO_3_ and SrIrO_3_ have complicated distribution of band-anti-crossings in their band structures^22, 34, 35^, the quantitative evaluation of the electric field-induced modulation in the heterostructures requires elaborate theoretical investigation. Nevertheless, the present observations clearly indicate that the electric field applied to the thin SrIrO_3_ plays the crucial role in SOC at the interface and even brings about the significant modification of magnetic properties in the neighboring itinerant ferromagnet. The method of inserting a thin nonmagnetic material with strong SOC between a ferromagnet and a gate dielectric may be applicable to tuning many intriguing spin-orbit coupled phenomena such as magnetic anisotropy^15^, domain wall motion^36^ and the DMI iteself^37^.

## Methods

### Sample preparation

The epitaxial bilayers composed of SrIrO_3_ and SrRuO_3_, and single layer films of SrRuO_3_ were deposited on SrTiO_3_(001) substrates by pulsed laser deposition using a KrF excimer laser (*λ* = 248 nm). The substrate temperatures during the growth of SrRuO_3_ and SrIrO_3_ were 730 °C and 600 °C, respectively, where oxygen partial pressure was 120 mTorr. The laser fluence was 1.2 J/cm^2^ for SrRuO_3_ and 2.6 J/cm^2^ for SrIrO_3_.

### Measurement of magnetic and transport properties

The magnetization data were recorded by a SQUID magnetometer with a magnetic field applied perpendicularly to the film plane and along the magnetic easy axis grown on SrTiO_3_(001). Magneto-optic Kerr effect was measured with a laser at 690 nm wavelength in polar geometry by using a photoelastic modulator.

Transport properties were measured in Hall bars cut by a diamond wheel saw (1 mm × 2.5 mm) and ultrasonically bonded with Al wires. The applied current was 10 μA, which corresponds to 3.6 × 10^6^ A/m^2^ for SRO5/SIO2/Sub. Back-gate transistors were fabricated using 0.5-mm-thick SrTiO_3_ substrates as a gate dielectric and silver paste as a gate electrode at the opposite side of the deposited films. Antisymmetrizations were performed for both the Hall resistivity and the Kerr rotation angle. Ordinary Hall term was subtracted from the Hall resistivity by linear fitting in a higher magnetic field region.

### Data availability

The data that support the findings of this study are available from the corresponding author upon request.

## Electronic supplementary material

Supplementary Information
