# Supplementary material for: Electric-field control of anomalous and topological Hall effects in oxide bilayer thin films
Source: Nat Commun. 2018 Jan 15;9:213. doi: 10.1038/s41467-017-02629-3 (PMC5768777; doi:10.1038/s41467-017-02629-3)
Supplement: Supplementary file 1 — Supplementary Information [file 41467_2017_2629_MOESM1_ESM.pdf]

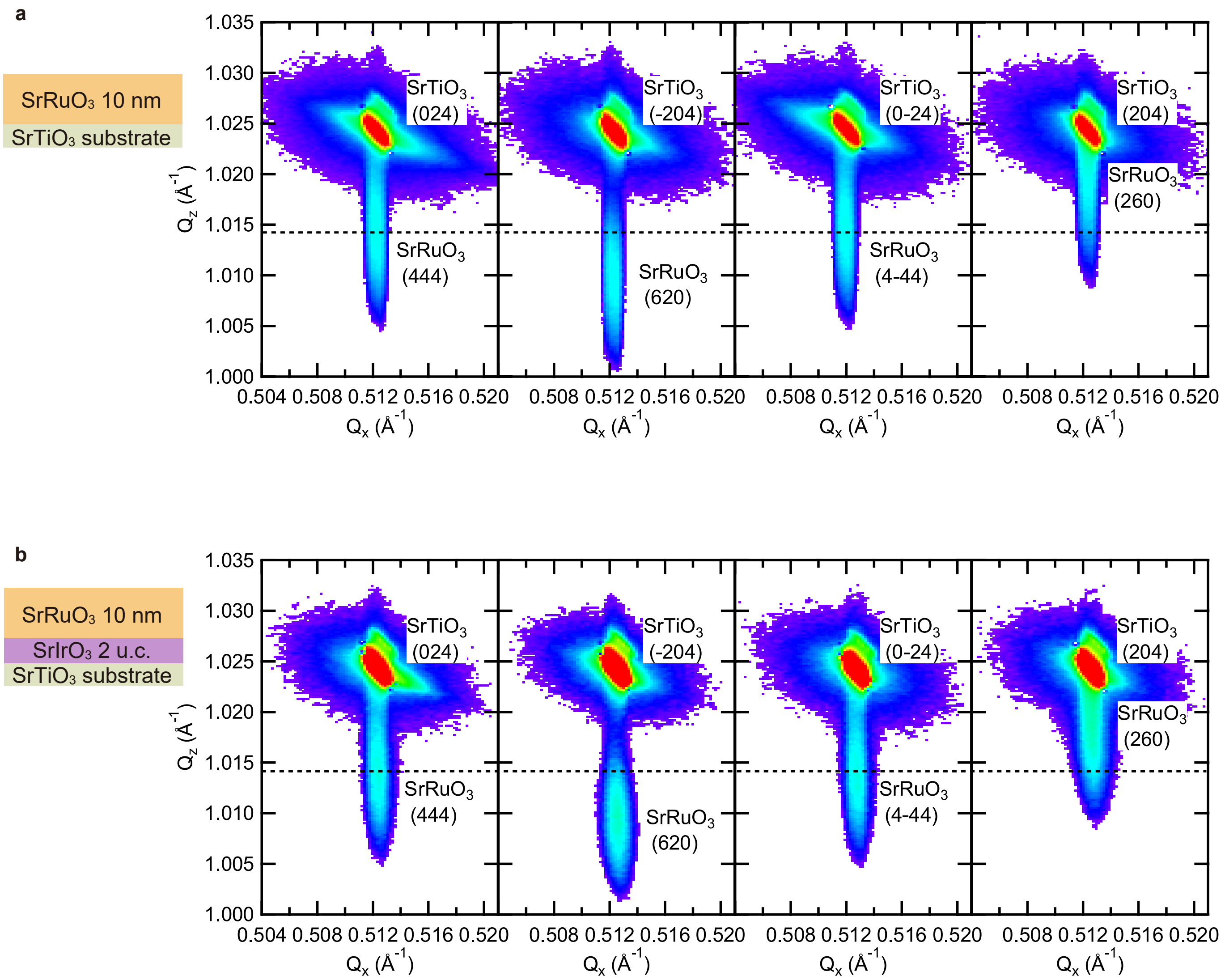

Supplementary Figure 1 | Reciprocal space maps of $SrRuO_3$ films without/with $SrIrO_3$ insertion

Reciprocal space maps of 10-nm-thick $SrRuO_3$ grown on (**a**) a $SrTiO_3$(001) substrate and (**b**) those with the $SrIrO_3$ insertion layer. The respective structure is shown in left.

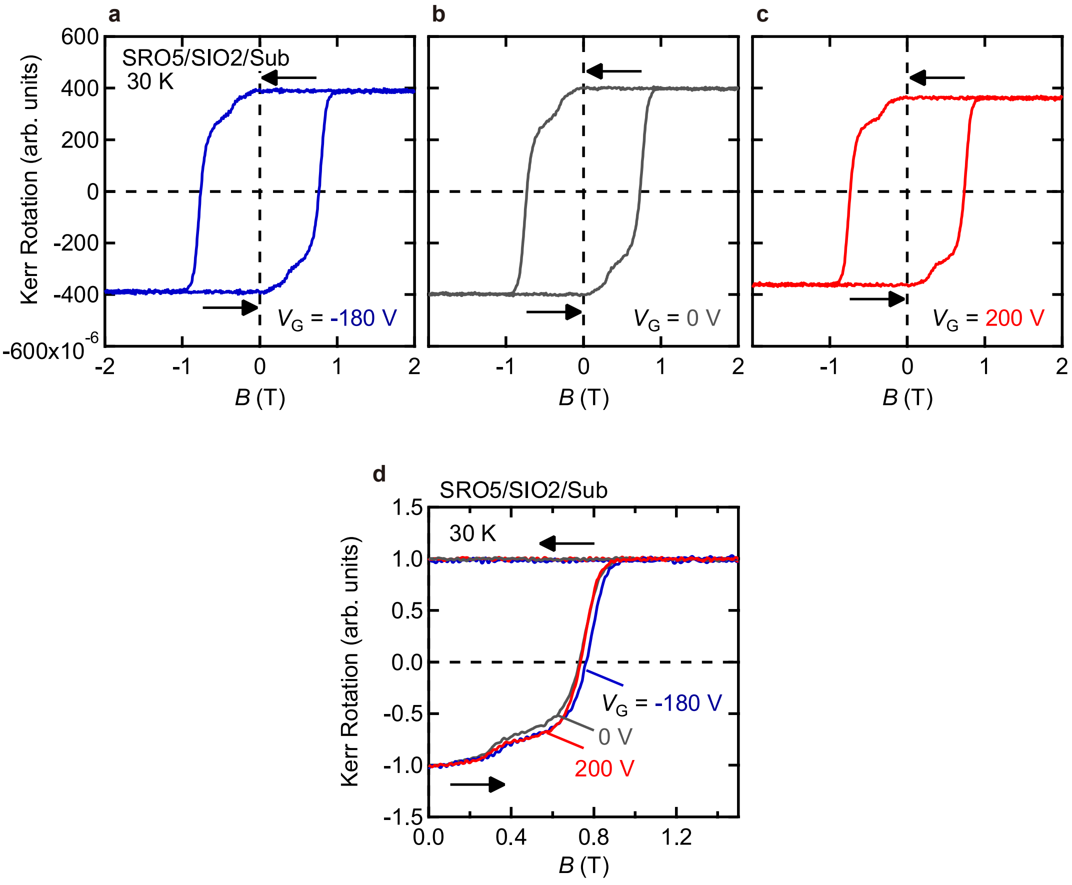

Supplementary Figure 2 | Magneto-optic Kerr rotation as a function of applied electric field

Magnetic-field (*B*) dependence of Kerr rotation at 30 K under gate voltage $V_G = -180$ V (**a**), 0 V (**b**), and 200 V (**c**) in SRO5/SIO2/Sub. The data are not normalized by ρ_{AHE} in contrast to those shown in Figs. 2**a-c** in the main text. Black arrows represent field-sweep directions. **d,** The same Kerr rotation under $V_G = -180$ V (blue), 0 V (grey), and 200 V (red) normalized by the each saturated value at 2 T.

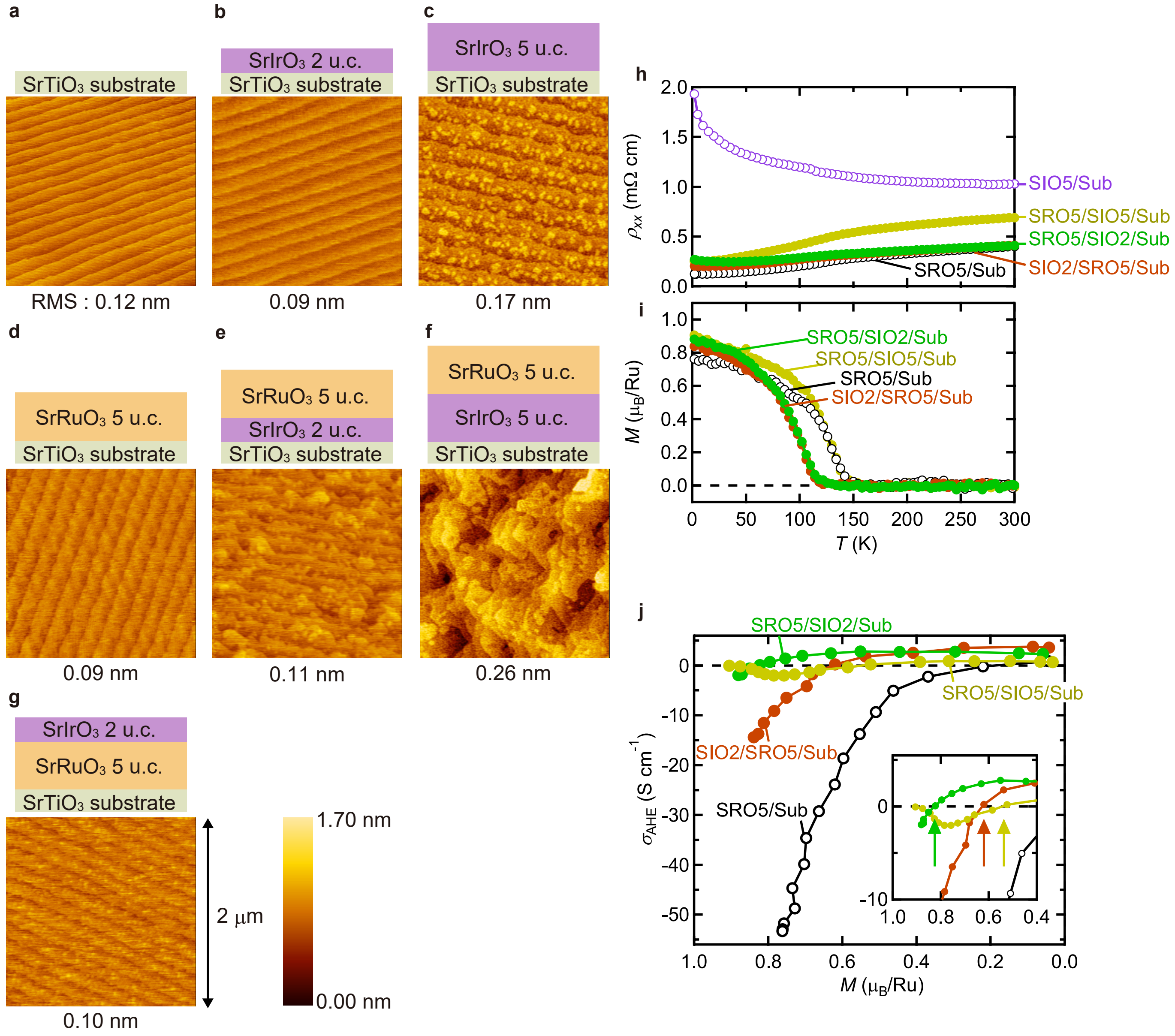

Supplementary Figure 3 | Basic physical properties of $SrRuO_3$-$SrIrO_3$ heterostructures with different $SrIrO_3$ thickness

AFM images of the surface morphology for films with varied $SrIrO_3$ thickness (0, 2, 5 unit cells) on $SrTiO_3$ substrates (**a-c**) and those with a 5-unit-cell $SrRuO_3$ top layer (**d-f**). The image of 2-unit-cell $SrIrO_3$ on 5-unit-cell $SrRuO_3$ is shown for comparison (**g**). Sample structure and Root-Mean-Square (RMS) roughness are shown above and below the images, respectively. Temperature (T) dependence of longitudinal resistivity (ρ_{xx}, **h**) and out-of-plane magnetization measured at 0.1 T (M, **i**). **j**, Anomalous Hall conductivity (σ_{AHE}) as a function of M. Inset shows the magnified view around the sign reversal of σ_{AHE} indicated by arrows.

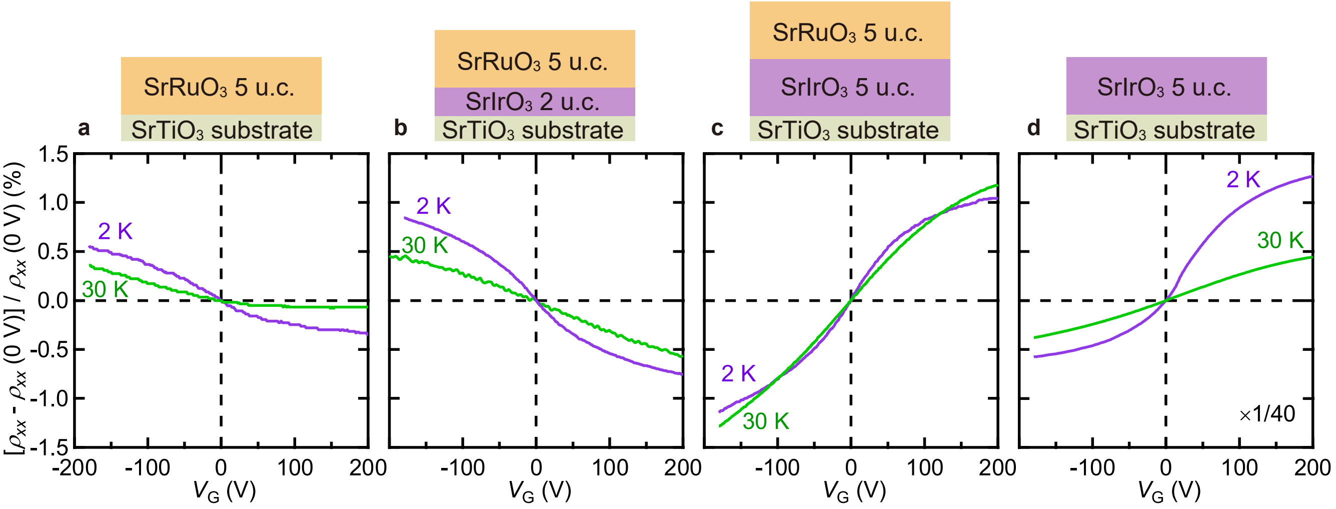

Supplementary Figure 4 | V_G dependence of longitudinal resistivity

a-d, Longitudinal resistivity variation divided by the zero-bias value $[\rho_{xx} - \rho_{xx}\,(0\ \mathrm{V})]/\rho_{xx}\,(0\ \mathrm{V})$ as a function of gate voltage (V_G) at 2 K and 30 K. On top, respective sample structure is shown.

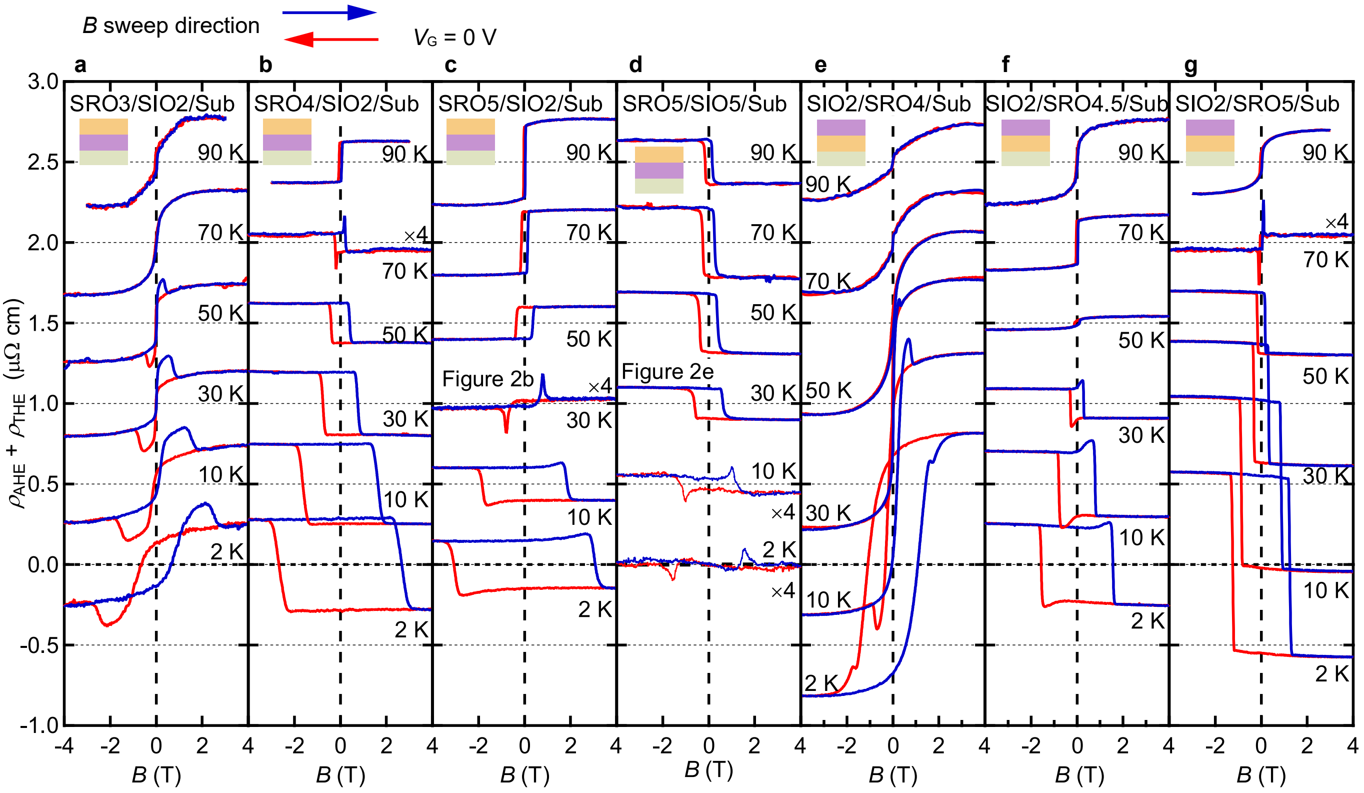

Supplementary Figure 5 | Magneto-transport properties in heterostructures of $SrIrO_3$ and $SrRuO_3$

Magnetic-field (*B*) dependence of anomalous and topological Hall resistivities ($\rho_{AHE} + \rho_{THE}$) in SRO*m*/SIO2/Sub [*m* = 3 (**a**), 4 (**b**), and 5 (**c**) unit cells], SRO5/SIO5/Sub (**d**) and SIO2/SRO*m*/Sub [*m* = 4 (**e**), 4.5 (**f**), and 5 (**g**) unit cells] at various temperatures. Stacking sequence is displayed in inset of the each panel. Red (blue) curve corresponds to downward (upward) sweep of *B*. All measurements were performed under gate voltage V_G = 0 V.

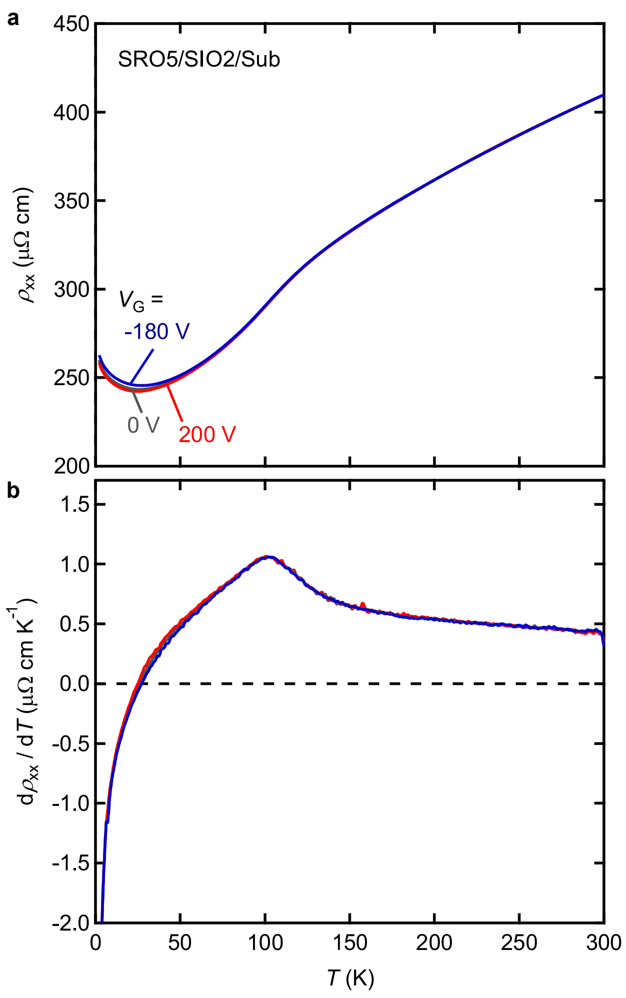

Supplementary Figure 6 | Negligible Curie temperature variation by applied electric field

Temperature (T) dependence of longitudinal resistivity (ρ_{xx}, **a**) and differential resistivity ($d\rho_{xx}$ / dT, **b**) under gate voltage V_G = −180 V (blue), 0 V (grey), and 200 V (red) in SRO5/SIO2/Sub.

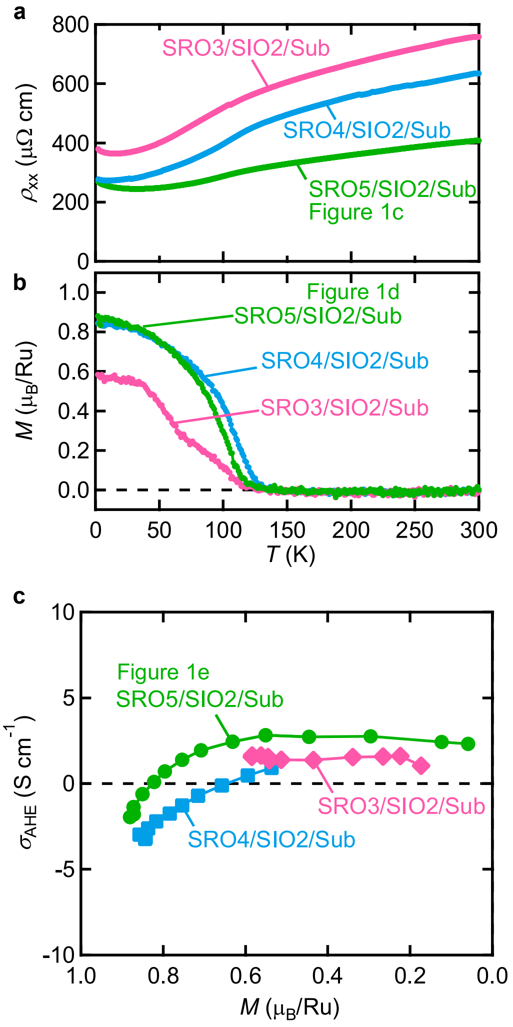

Supplementary Figure 7 | Basic physical properties of $SrRuO_3/SrIrO_3/SrTiO_3$ with different $SrRuO_3$ thickness

Temperature (T) dependence of longitudinal resistivity (ρ_{xx}, **a**) and out-of-plane magnetization measured under 0.1 T (M, **b**) in SROm/SIO2/Sub (m = 3, 4, and 5 unit cells). **c**, Anomalous Hall conductivity (σ_{AHE}) as a function of magnetization (M).

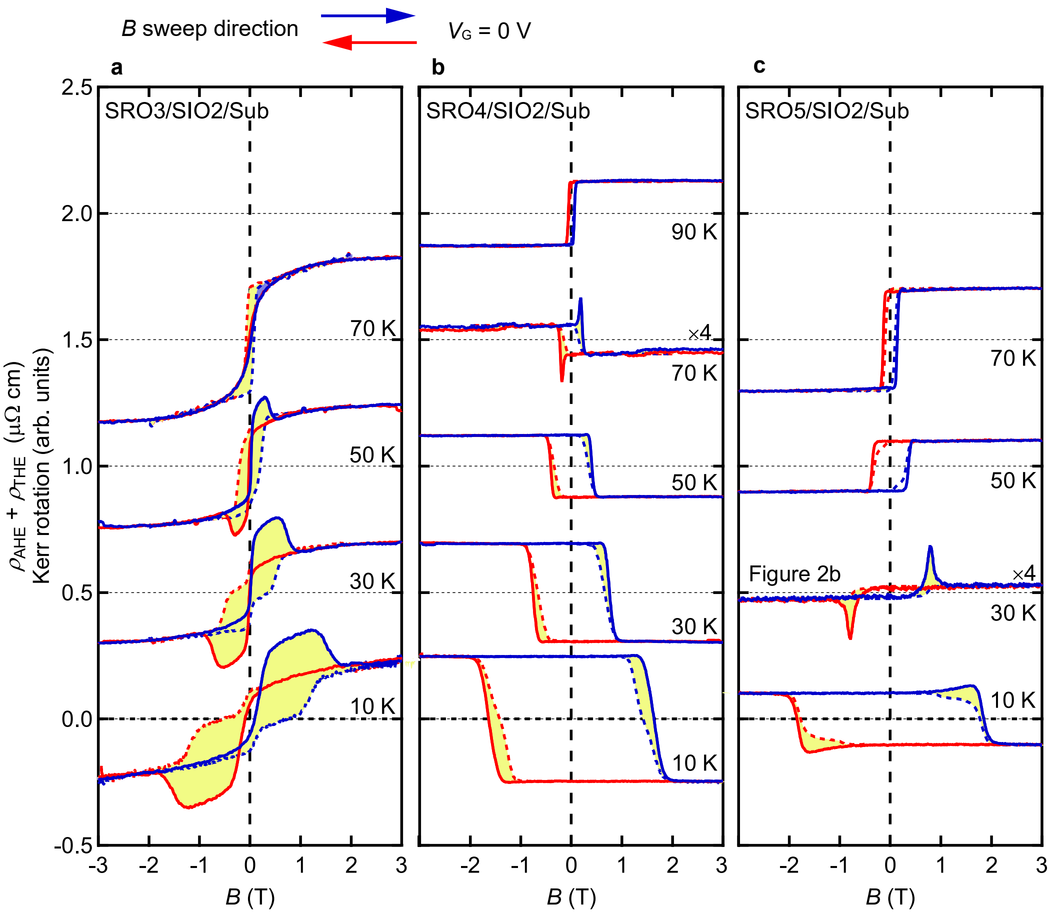

Supplementary Figure 8 | Topological Hall effect deduced from transport and magneto-optic Kerr effect measurements in SRO*m*/SIO2/Sub (*m* = 3, 4, and 5)

Magnetic-field (B) dependence of anomalous and topological Hall resistivitites (ρ_{AHE} + ρ_{THE}, solid lines) under gate voltage V_G = 0 V for SRO*m*/SIO2/Sub [m = 3 (**a**), 4 (**b**), and 5 (**c**)]. Kerr rotations are also shown by broken lines. Yellow coloured regions correspond to topological Hall resistivity (ρ_{THE}). Red (blue) curves correspond to downward (upward) sweeps of B.

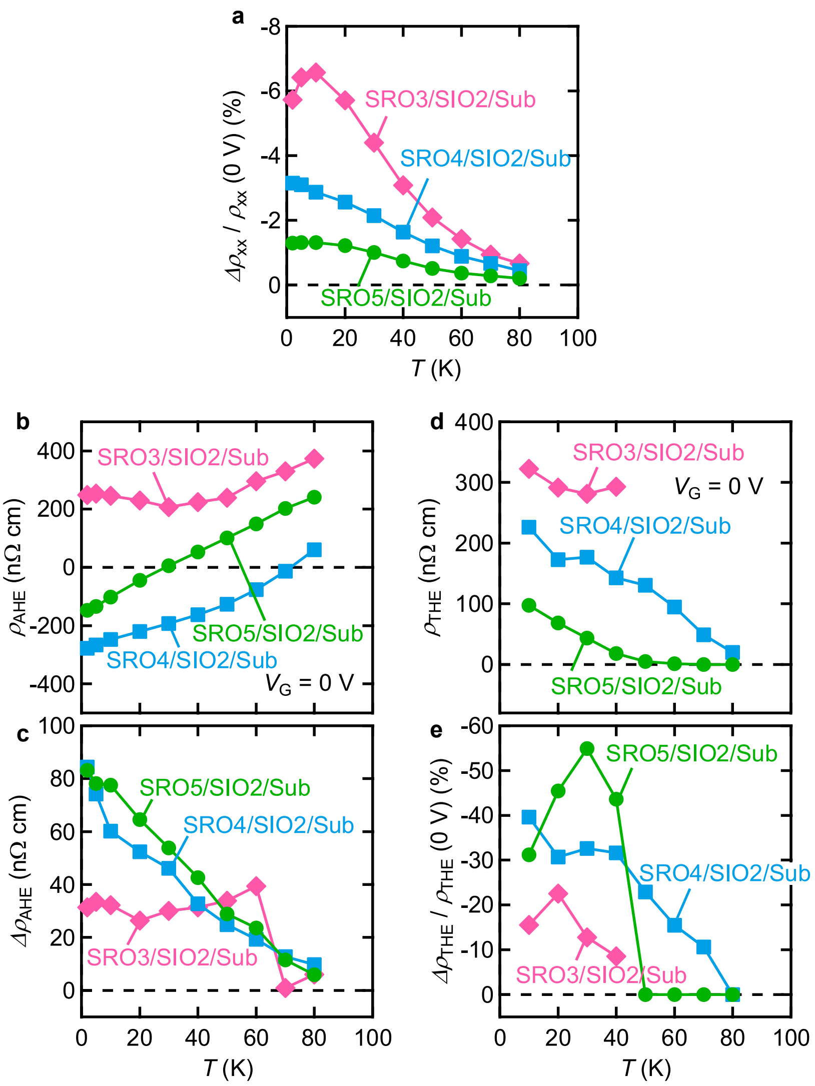

Supplementary Figure 9 | Temperature dependence of transport properties and their gate modulations in SRO*m*/SIO2/Sub (*m* = 3, 4, and 5)

a, Temperature (T) dependence of variation in longitudinal resistivity normalized by the zero-bias ($V_G = 0$ V) value [$\Delta\rho_{xx}/\rho_{xx}$ (0 V)]. T dependence of anomalous Hall resistivity (ρ_{AHE}, **b**), and its modulation amplitude [$\Delta\rho_{AHE} = \rho_{AHE}$ ($V_G = 200$ V) − ρ_{AHE} ($V_G = -180$ V), **c**]. T dependence of topological Hall resistivity (ρ_{THE}, **d**), and its modulation amplitude normalized by zero-bias value [$\Delta\rho_{THE}/\rho_{THE}$ (0 V), **e**].

Supplementary Note 1 | Lattice structure of $SrIrO_3$-inserted $SrRuO_3$

It has been reported that $SrRuO_3$ has the structural deformation from its original orthorhombic structure to tetragonal one due to the octahedral rotation mismatch with a buffer layer on a $SrTiO_3$ substrate[1], accompanying the modification of magnetic anisotropy. In our case of $SrRuO_3$/$SrIrO_3$/$SrTiO_3$, this kind of structural phase change might be triggered since $SrIrO_3$ can serve as a buffer layer. However, in the structural point of view, the insertion of $SrIrO_3$ is expected to have negligible effect on the structural properties of the top $SrRuO_3$ layer. The large structural distortion in the heterointerface of perovskite oxides is known to be induced by the combinations with a large difference in ionic radii, because this leads to a large difference in the distortion of the octahedra formed with *B*-site transition metals and surrounding oxygens. In our case, the ionic radius of Ru^{4+} is about 62 pm, which is close to that of Ir^{4+} (62.5 pm).

In fact, as shown in the x-ray diffraction data in Supplementary Figs. 1a and 1b, we confirmed that the 10-nm-thick $SrRuO_3$ films have the same orthorhombic (or lower symmetry) structure at room temperature regardless of the insertion of 2-unit-cell $SrIrO_3$ on $SrTiO_3$ substrates, indicating $SrIrO_3$ has a negligible impact on the lattice structure of $SrRuO_3$. Furthermore, as shown in Fig. 1d in the main text, the ferromagnetic properties such as Curie temperature (T_C) and the value of magnetization are quite similar between SRO5/SIO2/Sub and SIO2/SRO5/Sub. These similarities are in agreement with the identical lattice structure although we cannot resolve the reciprocal space maps in such thin heterostructures. Therefore we conclude that the stacking order brings a negligible impact on the structural details of $SrRuO_3$.

Supplementary Note 2 | Magneto-optic Kerr effect (MOKE) under applied electric fields

In Supplementary Figs. 2a-c, we show magnetic-field dependences of Kerr rotation in SRO5/SIO2/Sub at 30 K under different gate biases (V_G). The sign of all measurements at saturation field is the same with each other in spite of the sign inversion of anomalous Hall resistivity (ρ_{AHE}). This fixed sign of Kerr rotation indicates that the magnetization itself is not inverted by the applied electric field, confirming the variation of the proportionality factor R_S in the intrinsic anomalous Hall effect (AHE). We note that the saturated Kerr rotation under each applied electric field has a limited accuracy; this fluctuation is at most 10% of the saturated values at V_G = 0 V. Hence, the electric-field-induced variation of magnetization is under this order of magnitude. The same Kerr rotations normalized by each saturated value at 2 T are shown in Supplementary Fig. 2d. The coercive force (H_C) is shifted from 0.76 T (V_G = 200 V) to 0.73 T (V_G = −180 V) at 30 K; this shift of H_C implies the modulation of spin-orbit coupling (SOC) because the magnetic anisotropy results from SOC.

Supplementary Note 3 | Thickness dependence of $SrIrO_3$ in $SrRuO_3/SrIrO_3/SrTiO_3$

In Supplementary Figs. 3a-c, we show atomic force microscopy (AFM) images of the surface morphology for the $SrIrO_3$ films grown on $SrTiO_3$(001) substrates (SIO*x*/Sub, *x* = 0, 2, 5 unit cells, respectively). The clear step-and-terrace structure starts to be degraded in SIO5/Sub, while the structure is kept in SIO2/Sub. This thickness dependence of the surface morphology results from the lack of step-flow growth in SIO*x*/Sub when *x* is larger than 2. Supplementary Figures 3d-f show AFM images of SRO5/Sub, SRO5/SIO2/Sub and SRO5/SIO5/Sub respectively. In accord with the trend

of SIOx/Sub, the heterostructure with thicker $SrIrO_3$ (SRO5/SIO5/Sub) has the worse surface flatness, where the step-and-terrace structure almost disappears. The root-mean-square (RMS) roughness is 0.26 nm, which deviates from the ideal value of a flat step-and-terrace surface of $SrRuO_3$ (0.11 nm). In Supplementary Fig. 3g, we show the preserved step-and-terrace surface morphology of SIO2/SRO5/Sub for comparison.

Supplementary Fig. 3h shows temperature dependence of longitudinal resistivity (ρ_{xx}) in the samples with 5-unit-cell $SrRuO_3$. For comparison, we plot ρ_{xx} in SIO5/Sub, indicating the semimetallic temperature dependence as reported in the thicker films[2,3]. Due to the finite contribution of the $SrIrO_3$ layer, ρ_{xx} in SRO5/SIO5/Sub is higher than those in other samples at room temperature, while the conductivity in the $SrRuO_3$ layer is dominant at lower temperature. Supplementary Fig. 3i shows temperature dependence of magnetization in the same set of the samples. The magnetization at 2 K in SRO5/SIO5/Sub has almost the same value with those of the other samples, suggesting nearly the same thickness of $SrRuO_3$. Supplementary Figure 3j shows anomalous Hall conductivity (σ_{AHE}) as a function of magnetization (M). The sign of σ_{AHE} in SRO5/SIO5/Sub is reversed roughly at the value of 0.6 μ_{B}/Ru, which is close to those in the other samples as indicated by arrows in the inset of Supplementary Fig. 3j.

The influence of the thicker $SrIrO_3$ is also observed in V_{G} dependence of ρ_{xx} as well as the suppressed electrical control of AHE mentioned in the main text. Supplementary Figures 4a-d show longitudinal resistivity variations $[\rho_{xx} - \rho_{xx}\,(0\ \mathrm{V})]/\rho_{xx}\,(0\ \mathrm{V})$ as a function of V_{G} at low temperatures. In the thinner $SrIrO_3$ sample (SRO5/SIO2/Sub, Supplementary Fig. 4b), the V_{G} dependence shows the behaviour of electron systems, which is the same with that in SRO5/Sub

(Supplementary Fig. 4a). By contrast, in the thicker $SrIrO_3$ sample (SRO5/SIO5/Sub, Supplementary Fig. 4c), the V_G dependence shows the behaviour of hole systems, which is the same with that in SIO5/Sub (Supplementary Fig. 4d). The change in modulated carrier type indicates that most of the electric field is screened within the $SrIrO_3$ layer to give the hole-type carrier density modulation, resulting in the smaller electric-field effect to the SRO/SIO interface in SRO5/SIO5/Sub. This is consistent with the smaller variation in anomalous Hall resistivity as shown in Fig. 2f in the main text.

Supplementary Note 4 | Magneto-transport properties in all heterostructures

In Supplementary Figs. 5a-g, we show the sum of anomalous and topological Hall resistivities (ρ_{AHE} + ρ_{THE}) at various temperatures as a function of magnetic field in all examined heterostructures, where the thickness of $SrRuO_3$ ranges from 3 to 5 unit cells. In this thickness region of $SrRuO_3$, the heterostructures exhibit topological Hall effect (THE) as an additional term deviated from the conventional ferromagnetic hysteresis curve of AHE regardless of the stacking sequence of $SrRuO_3$ and $SrIrO_3$. Both the sign of AHE and the appearance of THE in SIO2/SRO*m*/Sub are qualitatively the same with the previous report[4] dealing with the same stacking order. We note that AHE with inverted structures (SRO*m*/SIO2/Sub) indicates a slight difference from the reported structures (SIO2/SRO*m*/Sub). When we look at 4-unit-cell $SrRuO_3$ samples (SRO4/SIO2/Sub and SIO2/SRO4/Sub), for example, the sign inversion of AHE occurs in SRO4/SIO2/Sub while it does not in SIO2/SRO4/Sub, exhibiting positive sign of the AHE in all measured temperatures. This difference is probably ascribed to the slight discordance of chemical potential or electron localization, which may come from some distortions in crystals depending on the stacking sequence.

Supplementary Note 5 | Negligible T_C variation by applied electric field

In Supplementary Figs. 6a and 6b, we show temperature dependences of ρ_{xx} and dρ_{xx} / dT under different electric fields in SRO5/SIO2/Sub. Since the ferromagnetic transition in $SrRuO_3$ is accompanied by a kink structure of ρ_{xx}, we can estimate the electric-field-induced T_C variation from the anomaly in ρ_{xx}, which is observed as the increase in dρ_{xx} / dT. However, the observed dρ_{xx} / dT is almost unchanged by electric field, suggesting the negligible shift of T_C. This V_G-independent behaviour of T_C implies that the ferromagnetic interaction J is almost unvaried by the applied electric field because the change of J should contribute to the shift of T_C.

Supplementary Note 6 | Basic transport properties in $SrRuO_3$/$SrIrO_3$/$SrTiO_3$

In Supplementary Figs. 7a-c, we show basic transport properties in SRO*m*/SIO2/Sub (m = 3, 4, and 5 unit cells). It is known that high-quality $SrRuO_3$ films on atomically flat $SrTiO_3$(001) substrates exhibit metallic conduction and ferromagnetism until the thickness is reduced to approximately 3 unit cells[5,6]. However, it is not straightforward to grow such a thin itinerant ferromagnetic $SrRuO_3$ layer on a $SrIrO_3$ layer, even though the 2-unit-cell $SrIrO_3$ seems atomically flat according to its surface morphology as shown in Supplementary Fig. 3b. By carefully optimizing growth conditions, our heterostructures composed of $SrRuO_3$ and $SrIrO_3$ maintain their itinerant ferromagnetic temperature dependences (Supplementary Fig. 7a) until the thickness of $SrRuO_3$ reaches the thin limit of 3 unit cells.

When $SrRuO_3$ layer thickness is reduced, the longitudinal resistivity is enhanced (Supplementary Fig. 7a). At the same time, ferromagnetic Curie temperature and magnetization normal to the film plane are suppressed compared with those of bulk[7]

(T_C ~ 160 K, M ~ 1.6 μ_B/Ru) as shown in Supplementary Fig. 7b. These systematic suppressions of the itinerant ferromagnetic natures are attributed to electron localization in $SrRuO_3$ and are also observed in other ultrathin $SrRuO_3$ films[5,6]. As a result, anomalous Hall conductivity (σ_{AHE}) shows no sign inversion in SRO3/SIO2/Sub (Supplementary Fig. 7c), which is consistent with the scaling character of σ_{AHE} as a function of M. The thin $SrIrO_3$ layer likely contributes to electron conduction, while the 2-unit-cell $SrIrO_3$ alone appears to be insulating with a resistivity over 10 Ω cm at room temperature.

Supplementary Note 7 | Topological Hall effect in $SrRuO_3$/$SrIrO_3$/$SrTiO_3$

Finite THE is extracted from magneto-transport ($\rho_{AHE} + \rho_{THE}$) and MOKE measurements in SRO*m*/SIO2/Sub (m = 3 and 4) (Supplementary Figs. 8a and 8b) as well as that in m = 5 (Supplementary Fig. 8c) as already shown in Fig. 2b in the main text. In all measurements, the magnetic fields at which hysteresis curves are closed in MOKE measurements approximately coincide with those in magneto-transport measurements. This indicates that the both measurements were properly performed at almost the same temperature. Kerr rotations normalized by $\rho_{AHE} + \rho_{THE}$ at those magnetic fields represent the anomalous Hall component because both measurements represent only ferromagnetic magnetization. At higher temperatures in SRO3/SIO2/Sub, however, the magneto-transport properties cease to represent pure AHE and THE because the nature of multicarrier in $SrRuO_3$ appears in Hall effect near T_C. As a result, we were not able to deduce the reliable value of THE in this sample at 50 K and more.

Supplementary Note 8 | Temperature dependence of electrical modulations in $SrRuO_3/SrIrO_3/SrTiO_3$

In Supplementary Figs. 9a-e, we show the series of magneto-transport properties and their electric-field modulations from which we deduce Fig. 4 in the main text. Since ρ_{xx} is inversely proportional to carrier density (n), the observed $\Delta\rho_{xx}/\rho_{xx}$ (0 V) is consistent with the expected electron accumulation by a $SrTiO_3$ back-gate transistor. At 10 K, for example, the measured electron density was 2×10^{15} cm^{-2} (1×10^{22} cm^{-3} in 5 unit cells of $SrRuO_3$) under V_G = 0 V. Meanwhile, the accumulated sheet carrier density is expected to be about 1×10^{13} cm^{-2} under V_G = 200 V, which corresponds to 0.5% decrease in ρ_{xx}, that is, approximately the half value of the observed difference between plus (200 V) and minus (−180 V) gate bias. Therefore, we were roughly able to regard $\Delta\rho_{xx}/\rho_{xx}$ (0 V) as the modulation of carrier density. In all measurements of the electric-field effect, all of the transport properties (ρ_{xx}, ρ_{AHE}, and ρ_{THE}) under V_G = 0 V are located between those under V_G = 200 V and V_G = −180 V. The ρ_{THE} gets larger as the thickness of $SrRuO_3$ gets thinner as shown in Supplementary Fig. 9d. This thickness dependence of ρ_{THE} is consistent with the previous report in the structure of SIO2/SRO*m*/Sub[4].

Supplementary References

1. Gao, R. *et al.* Interfacial Octahedral Rotation Mismatch Control of the Symmetry and Properties of $SrRuO_3$. *ACS Appl. Mater. Interfaces* **8,** 14871–14878 (2016).
2. Wu, F.-X. *et al.* Metal-insulator transition in $SrIrO_3$ with strong spin-orbit interaction. *J. Phys. Condens. Matter* **25,** 125604 (2013).
3. Matsuno, J. *et al.* Engineering a Spin-Orbital Magnetic Insulator by Tailoring Superlattices. *Phys. Rev. Lett.* **114,** 247209 (2015).
4. Matsuno, J. *et al.* Interface-driven topological Hall effect in $SrRuO_3$-$SrIrO_3$ bilayer. *Sci. Adv.* **2,** e1600304 (2016).
5. Xia, J., Siemons, W., Koster, G., Beasley, M. R. & Kapitulnik, A. Critical thickness for itinerant ferromagnetism in ultrathin films of $SrRuO_3$. *Phys. Rev. B* **79,** 140407(R) (2009).
6. Chang, Y. J. *et al.* Fundamental thickness limit of itinerant ferromagnetic $SrRuO_3$ thin films. *Phys. Rev. Lett.* **103,** 057201 (2009).
7. Cao, G., McCall, S., Shepard, M., Crow, J. E. & Guertin, R. P. Thermal, magnetic, and transport properties of single-crystal $Sr_{1-x}Ca_xRuO_3$ $(0<\sim x<\sim 1.0)$. *Phys. Rev. B* **56,** 321–329 (1997).
